# Using Genetically Encoded Calcium Indicators to Study Astrocyte Physiology: A Field Guide

**DOI:** 10.3389/fncel.2021.690147

**Published:** 2021-06-11

**Authors:** Christian Lohr, Antonia Beiersdorfer, Timo Fischer, Daniela Hirnet, Natalie Rotermund, Jessica Sauer, Kristina Schulz, Christine E. Gee

**Affiliations:** ^1^Division of Neurophysiology, University of Hamburg, Hamburg, Germany; ^2^Institute of Synaptic Physiology, University Medical Center Hamburg-Eppendorf, Hamburg, Germany

**Keywords:** glial cells, astrocyte, calcium imaging, genetically encoded calcium indicator, GCaMP, calcium sensor

## Abstract

Ca^2+^ imaging is the most frequently used technique to study glial cell physiology. While chemical Ca^2+^ indicators served to visualize and measure changes in glial cell cytosolic Ca^2+^ concentration for several decades, genetically encoded Ca^2+^ indicators (GECIs) have become state of the art in recent years. Great improvements have been made since the development of the first GECI and a large number of GECIs with different physical properties exist, rendering it difficult to select the optimal Ca^2+^ indicator. This review discusses some of the most frequently used GECIs and their suitability for glial cell research.

## Introduction

Astrocytes have long been considered as passive cells, merely supporting neurons and maintaining nervous tissue homeostasis. Physiological studies during the past two decades have fundamentally changed this view. Not only are astrocytes responsive to neuronally derived neurotransmitters, they also release signaling molecules named “gliotransmitters” to affect neuronal development and synaptic transmission (Clarke and Barres, 2013; Khakh and Sofroniew, 2015; Verkhratsky and Nedergaard, 2018; Kofuji and Araque, 2021a). Ca^2+^ signaling plays a pivotal role in both the physiology and function of astrocytes (Khakh and McCarthy, 2015; Semyanov et al., 2020). Therefore, it is not surprising that Ca^2+^ imaging is the main technique used to study astrocyte physiology (Lohr and Deitmer, 2010). While chemical Ca^2+^ indicators were employed in the early years to visualize Ca^2+^ signals in astrocytes, genetically encoded Ca^2+^ indicators (GECIs) have become the method of choice in state-of-the-art experimentation (Okubo and Iino, 2020; Yu et al., 2020). This review provides an overview of available Ca^2+^ indicators and compares their advantages and drawbacks to aid scientists in selecting the optimal GECI for glial cell research.

## Chemical Ca^2+^ Indicators

Since glial cells such as astrocytes are electrically non-excitable cells, electrophysiological techniques revealed only limited insights into astrocyte properties. Rather than changes in membrane potential, most astrocytic transmitter receptors cause a rise in cytosolic Ca^2+^ concentration (Deitmer et al., 1998; Kofuji and Araque, 2021b). The development of chemical fluorescent Ca^2+^ indicators, with a major contribution of Nobel laureate Roger Tsien, was a breakthrough for glial cell research (Tsien, 1989). Chemical Ca^2+^ indicators are fluorescent dyes that change their spectral properties upon binding of Ca^2+^ and therefore indicate changes in cytosolic Ca^2+^. As a keyword, Fura-2, the most popular chemical Ca^2+^ indicator, yields more than 12,000 hits in a Pubmed search, and its original publication (Grynkiewicz et al., 1985) has been cited over 21,000 times. The development of membrane-permeant acetoxymethyl ester (AM) derivatives of Fura-2 and other chemical Ca^2+^ indicators such as Fluo-3, Indo-1, and Calcium Green-1 coincided with the increasing availability of confocal fluorescence microscopy. This combination of circumstances resulted in a rapidly increasing number of studies of intracellular Ca^2+^ signaling in non-excitable cells, including astrocytes. Indicators with increased response amplitude, different spectral properties, and Ca^2+^ binding affinities, and the ability to be attached to membranes or accumulate in intracellular organelles were developed (Eberhard and Erne, 1991; Gee et al., 2000; Paredes et al., 2008). However, using chemical indicators to study astrocytes also has some drawbacks, in particular when applied in tissue such as brain slices. Although astrocytes are particularly efficient at taking up some AM dyes (Mulligan and MacVicar, 2004; Covelo and Araque, 2018; Tran et al., 2018), depending on brain region bulk-loading astrocytes with membrane-permeant Ca^2+^ indicators is not entirely cell-specific, hence neurons and other cells may also be loaded with the indicator (Singaravelu et al., 2006; Doengi et al., 2008; Lind et al., 2013; Otsu et al., 2015; Beiersdorfer et al., 2019). Consequently, astrocytes may need to be identified and distinguished from neurons. In many rodent brain areas, sulforhodamine 101 (SR101) is selectively taken up by astrocytes and can be used as a morphological marker of astrocytes (Nimmerjahn et al., 2004). In some brain regions, however, astrocytes fail to accumulate significant amounts of SR101 (Schnell et al., 2015) and SR101 has been reported to induce epileptic activity, limiting the applicability of SR101 (Kang et al., 2010). Other ways that can be employed to distinguish neuronal and astrocytic Ca^2+^ transients are to withdraw K^+^, which increases cytosolic Ca^2+^ in astrocytes but not neurons, as shown for brain stem, olfactory bulb, and cerebellum (Singaravelu et al., 2006; Härtel et al., 2007; Doengi et al., 2009; Fischer et al., 2020). In addition, we observed in the olfactory bulb that ATP induces Ca^2+^ transients in astrocytes and not in Fluo-4-loaded neurons (Doengi et al., 2008; Fischer et al., 2020, 2021). Hence, “physiological” markers can be used to distinguish between astrocytes and neurons when genetic or chemical markers cannot be applied. Somatic Ca^2+^ transients in astrocytes occur on a distinctively slower timescale than those in neurons, but this is not the case for transients in fine astrocytic processes and microdomains, which rise and fall on subsecond time scales. Recently, AM Ca^2+^ indicators and BAPTA AM have been shown to inhibit the Na^+^/K^+^ ATPase, compromising cellular metabolism and increasing extracellular K^+^, thus raising additional concerns about their use (Smith et al., 2018). Another disadvantage of bulk-loading astrocytes in brain slices with Ca^2+^ indicator dyes is a lack of contrast. If not only astrocytes but also other cells contain the dye, the surrounding tissue is bright and the very fine astrocyte processes do not stand out from the background. These problems are circumvented by loading a single astrocyte, e.g., by including the chemical Ca^2+^ indicator in a patch pipette used to record the astrocyte (Grosche et al., 1999; Henneberger et al., 2010). However, this procedure is time-consuming, requires additional equipment, and results in only a single dye-loaded astrocyte. First studies of single dye-loaded astrocytes revealed a hitherto unknown complexity of Ca^2+^ signaling in glial cells, including localized Ca^2+^ signals that occurred independently in very small microdomains (Grosche et al., 1999; Lohr and Deitmer, 1999; Di Castro et al., 2011). These results highlighted the need for improved methods to study astrocytic Ca^2+^ signaling in brain slices and *in vivo*.

## Genetically Encoded Ca^2+^ Indicators (GECIs)

The discovery that the gene encoding the green fluorescent protein (GFP) from *Aequoria victoria* can render other cells fluorescent heralded a new era in life science that included glial cell research (Prasher et al., 1992; Chalfie et al., 1994; Heim et al., 1995; Heim and Tsien, 1996). Protein engineering efforts produced fluorescent protein-based Ca^2+^ indicators by attaching the Ca^2+^ binding domains of calmodulin and the myosin light chain kinase peptides M13 and RS20, respectively, or troponin-C to the fluorescent proteins (Nakai et al., 2001; Heim and Griesbeck, 2004). The binding of Ca^2+^ to the Ca^2+^ binding domain then changes the conformation and spectral properties of the attached fluorescent protein(s), yielding a genetically encoded Ca^2+^ indicator (GECI) that can be expressed in genetically defined cells. GECIs have been employed to study glial Ca^2+^ signaling and proven superior compared to chemical Ca^2+^ indicators (Shigetomi et al., 2013).

Two principally different types of GECIs exist. Single wavelength GECIs consist of a Ca^2+^ sensing domain and a single fluorescent protein, whose fluorescence intensity changes when shifting between Ca^2+^-free and Ca^2+^-bound states, whereas FRET (Förster or fluorescence resonance energy transfer) GECIs consist of two fluorescent proteins linked by the Ca^2+^ binding domain (Mollinedo-Gajate et al., 2019; Inoue, 2020; Shen et al., 2020). Both types of GECIs have advantages and drawbacks and within each are many indicators with different properties. Selecting an indicator with appropriate spectral properties, Ca^2+^ binding affinity and dynamic range for the application can save a great deal of time and money. If it is important to quantify Ca^2+^ concentration, rather than Ca^2+^ dynamics, then FRET sensors, in which the energy transfer from donor to acceptor fluorophores change upon Ca^2+^ binding, are the option of choice. As the donor and acceptor fluorophores are expressed in a single protein, the ratio between the fluorescence intensity of the acceptor and the fluorescence intensity of the donor solely depends on Ca^2+^ concentration and is independent of expression levels. Stimulated emission/intensity FRET measurements may be affected, however, if fluorophore maturation or bleaching rates are very different, or when imaging deep in tissue because different wavelengths scatter differently. If using 2-photon excitation with wide-field detection, an additional problem arises: The dichroic mirrors in the detection pathway are sensitive to the angle of incident light and will split the light at different wavelengths, creating color gradients across the image. The resulting variations in red/green or cyan/yellow ratio across the field of view must be corrected for. Fluorescence lifetime imaging FRET (FLIM-FRET) of donor fluorescence is largely devoid of these artifacts but image acquisition is much slower and costly hybrid detectors and photon-counting boards are required. For quantification of Ca^2+^ signals with FRET sensors, the Ca^2+^ binding domain used to construct the GECI must also be considered. The Ca^2+^ binding domain of calmodulin, e.g., binds four calcium ions with positive cooperativity with a Hill coefficient in the range of 2, leading to a non-linear representation of the ambient Ca^2+^ concentration by the FRET ratio (Mank and Griesbeck, 2008). Recent FRET sensors of the Twitch family use a minimal Ca^2+^ binding motif of troponin C, which lacks cooperativity and therefore exhibits much better linearity of the Ca^2+^/FRET relationship (Thestrup et al., 2014; Wilms and Häusser, 2014).

**Figure 1:**
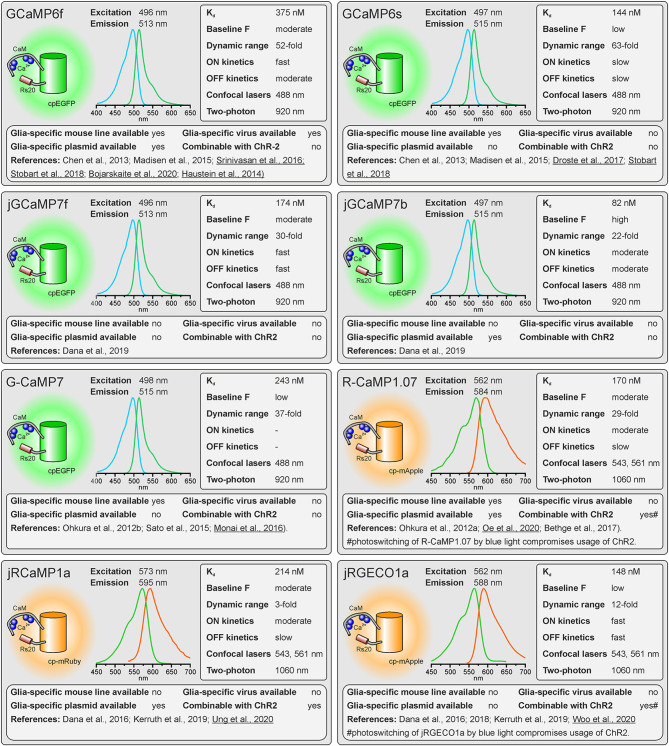
Properties of single-fluorophore Ca^2+^ sensors. See text for details. References using Ca^2+^ sensors in astrocytes are underlined.

The reason single wavelength GECIs are unsuitable for determining Ca^2+^ concentration is that intensity is not only Ca^2+^-dependent but depends critically on protein expression levels and imaging conditions. Single wavelength GECIs are, however, the option of choice for studying Ca^2+^ dynamics. Indeed, non-ratiometric GECIs such as GFP-based GCaMPs are the most popular Ca^2+^ indicators for studying glial cell physiology (Nakai et al., 2001; Ohkura et al., 2012b; Chen et al., 2013; Srinivasan et al., 2016; Droste et al., 2017; Stobart et al., 2018). Firstly, GCaMPs of the latest generations exhibit an enormous dynamic range, displaying a several 100 percent increase in fluorescence upon binding of Ca^2+^ (Figure 1). In addition, non-ratiometric GECIs require only one detector channel of the microscope to visualize Ca^2+^-dependent changes in fluorescence, leaving additional channels for other reporter proteins, fluorescent probes, and GECIs with different spectral properties to image Ca^2+^ changes, e.g., in a second cell type or multiple intracellular compartments. For instance, this method has been used to record Ca^2+^ signals in astrocytes and neurons with two spectrally different Ca^2+^ indicators or Ca^2+^ and cAMP simultaneously in cortical astrocytes using green fluorescent G-CaMP7 and red fluorescent Pink Flamindo, respectively (Stobart et al., 2018; Bojarskaite et al., 2020; Oe et al., 2020; Ung et al., 2020). FRET indicators, in contrast, occupy two detector channels. Since most FRET indicators comprise CFP and YFP (or their derivatives) as donor and acceptor proteins, light sources for excitation of around 430–450 nm (for CFP) and 515 nm (for YFP) are necessary and, hence, the configuration of the available microscopes needs to be checked before selecting such indicators. Whereas for the actual FRET measurement only the donor fluorophore needs to be excited, the ability to directly excite the acceptor is needed as well for setting up the experiments. One additional drawback of most FRET GECIs is their incompatibility with channelrhodopsin-2 (ChR2), the most frequently used optogenetic tool to stimulate neurons (Nagel et al., 2003; Boyden et al., 2005). ChR2 has a broad excitation spectrum, peaking at 450 nm. Thus, excitation of the FRET donor protein will inevitably activate ChR2 during the imaging process. This problem also occurs with green fluorescent non-ratiometric GECIs that are excited at 488 nm, however, several red fluorescent non-ratiometric GECIs with excitation peaks not interfering with ChR2 excitation are available.

Not only the properties of the fluorescent proteins determine the usefulness of the Ca^2+^ indicator, but also the second component, the Ca^2+^ binding domain. Mainly two different proteins provided Ca^2+^ binding domains for GECIs: Calmodulin and troponin C (Nakai et al., 2001; Heim and Griesbeck, 2004). In both cases, GECIs with fast kinetics and good dynamic range could be developed. However, GCaMPs using calmodulin have the drawback that many proteins interacting with calmodulin exist in glial cells and neurons and, hence, high expression of the GECI might interfere with calmodulin-dependent pathways in the cells unless modified (Yang et al., 2018). Troponin C, in contrast, is a protein only found in muscle cells and has no function in neural cells, avoiding undesirable effects of the Ca^2+^ indicator on cell physiology (Direnberger et al., 2012). Nevertheless, the troponin C-based Ca^2+^ indicator TN-XXL has also been shown to have detrimental side-effects leading to impaired neural development (Gasterstädt et al., 2020). Other Ca^2+^ binding proteins have been employed to construct GECIs that do not interfere with the biochemical environment in mammalian cells, including calmodulin derived from *Aspergillus* fungi in the FGCaMPs (Barykina et al., 2020). In addition to the biological activity of the Ca^2+^ binding domain, Ca^2+^ binding itself affects biological processes because the Ca^2+^ binding domains act as Ca^2+^ buffers that can significantly add to the endogenous Ca^2+^ buffer capacity. This is particularly perturbing using GECIs with high Ca^2+^ affinity (low K_d_) and multiple Ca^2+^ binding sites (four for most of the GECIs) and a key reason why expression levels must be kept low.

## A Field Guide to GECIs for Use in Glial Cells

**Figure 2:**
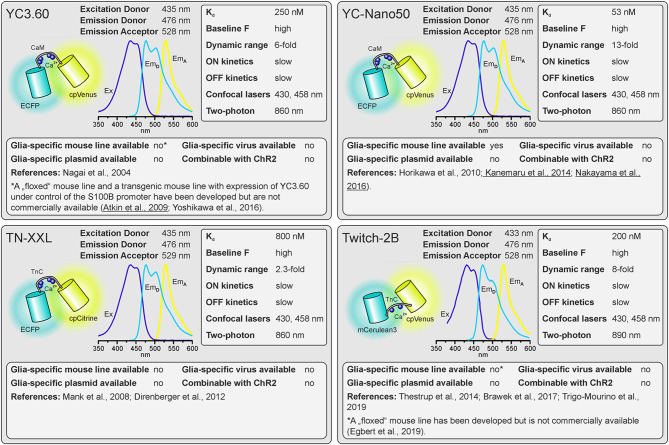
Properties of dual-fluorophore (FRET) Ca^2+^ sensors. See text for details. References using Ca^2+^ sensors in astrocytes are underlined. Ex, excitation; Em_D_, Emission of donor; Em_A_, Emission of acceptor.

As outlined above, there are many factors to consider when selecting the appropriate GECI. There are numerous available GECIs. Alone the derivatives descending from the first GCaMP developed by Nakai et al. (2001) comprise more than 50 members (Kerruth et al., 2019). In the following, we highlight the most popular GECIs *currently used* for glial cell research or related fields, their key properties, and for which applications they are best suited. Figure 1 contains single wavelength GECIs (Ohkura et al., 2012a; Chen et al., 2013; Dana et al., 2018, 2019) and Figure 2 GECIs suitable for ratiometric FRET and FLIM-FRET imaging (Nagai et al., 2004; Mank et al., 2008; Horikawa et al., 2010; Trigo-Mourino et al., 2019). While recently published GECIs are included, there are others not yet tested in astrocytes that will eventually prove superior. We, therefore, compare and discuss the properties that make particular GECIs useful for particular applications, which should help in selecting from yet newer GECIs and not only from those in Figures 1, 2. In the figurative summaries, we have highlighted those references in which GECIs have been published in astrocytes (Atkin et al., 2009; Haustein et al., 2014; Kanemaru et al., 2014; Monai et al., 2016; Nakayama et al., 2016; Srinivasan et al., 2016; Stobart et al., 2018; Woo et al., 2020).

### Spectral Properties

The first considerations are the excitation and emission spectra of the Ca^2+^ indicators. These must be matched to the light source, detectors, and filter sets available or new optical elements must be purchased. The excitation and emission maxima are listed for the various indicators. Since only the donor fluorophore is excited when using dual fluorophore (FRET) sensors, the excitation spectrum and maximum of the acceptor fluorophore are not quoted but it is highly advisable to also have the ability to directly excite the acceptor to visualize the cells, e.g., in order to select the optimal field of view.

### Dissociation Constant K_d_

The K_d_ reflects the Ca^2+^ concentration at which half of the Ca^2+^ indicator molecules are bound by Ca^2+^, while the other half is dissociated (Paredes et al., 2008). This also defines the point at which the relationship between an increase in the Ca^2+^ concentration and the resulting change in fluorescence is close to linear. The resting Ca^2+^ concentration in astrocytes is in the range of 80–100 nM (Deitmer et al., 1998), hence GECIs with K_d_ values close to this concentration will record Ca^2+^ changes reliably. However, if very large Ca^2+^ signals are expected with a peak concentration of 1 μM and above, the Ca^2+^ indicators might saturate and the amplitude of the Ca^2+^ signal might be underestimated. Ca^2+^ indicators with higher K_d_ values are more appropriate for these measurements.

### Baseline Fluorescence

The GECIs with the highest dynamic range often achieve this by extremely low fluorescence in the apo state, i.e., when no Ca^2+^ is bound. While at first glance this seems like an excellent feature, in practice it becomes almost impossible to visualize and focus on astrocytes expressing for instance the indicator GCaMP6s unless they are pre-stimulated. For astrocyte imaging, particularly when one wants to examine signals in the fine processes, it is, therefore, advantageous to choose single wavelength GECIs that are already fairly bright at baseline Ca^2+^ concentrations (100 nM). This is much less important for FRET sensors as one can always excite the acceptor directly to visualize and focus before switching to excite the donor for the actual ratiometric measurements.

### Dynamic Range

For single-fluorophore indicators, the dynamic range is calculated by the difference between the fluorescence intensity when Ca^2+^-saturated (F_max_) and the fluorescence intensity in Ca^2+^-free conditions (F_min_) divided by the fluorescence value in Ca^2+^-free conditions: (*F*_max_ − *F*_min_)/*F*_min_. For FRET sensors, it is similarly calculated by using the FRET ratio values instead of the fluorescence values. As mentioned above, while a high dynamic range is always desirable for single-wavelength GECIs it often reflects very low baseline fluorescence rather than very bright Ca^2+^-bound fluorescence. It is therefore important to consider not only the dynamic range but also how bright the sensor is at baseline. Whereas the best single wavelength sensors now have dynamic ranges of several hundred percent, the FRET sensors always appear inferior in this regard at first glance. In fact, a dynamic range of 40% is excellent for a FRET sensor.

### On and Off Kinetics

The rate of binding and unbinding of Ca^2+^ by the indicator molecule is reflected by values such as the half rise time (t_1/2_ rise) and half decay time (t_1/2_ decay). If binding and unbinding of Ca^2+^ occurs much slower than the actual increase and decrease in the Ca^2+^ concentration, the time course of the Ca^2+^ signal is distorted. Although it was believed for many years that Ca^2+^ transients in astrocytes rise and decay at slow rates and hence can readily be recorded with Ca^2+^ indicators with slow on and off kinetics, more recent studies showed that fast Ca^2+^ signals in astrocyte microdomains can occur at a time scale of tens of ms (Di Castro et al., 2011; Stobart et al., 2018). A direct comparison between GCaMP3 and GCaMP6f revealed a much faster and more reliable detection of Ca^2+^ transients in astrocyte microdomains by GCaMP6f, while global Ca^2+^ transients were equally well recorded by both indicators, illustrating the need for fast Ca^2+^ binding kinetics of the Ca^2+^ indicator for measurements in microdomains (Ye et al., 2017). Therefore, GECIs with fast on and off kinetics should be selected when recording fast and local Ca^2+^ transients. In addition, low expression levels of the GECI are necessary to minimize the effect of the Ca^2+^ buffer capacity that is added by the Ca^2+^ sensors on Ca^2+^ signal kinetics.

### Suitability for Standard Confocal and Two-Photon Microscopy

Confocal microscopy is the standard technique when imaging glial Ca^2+^ signaling in brain slices. While confocal microscopes, as available in virtually any life science institution, are typically equipped with a 488-nm laser (cyan excitation), a 543-nm or 568-nm laser (green excitation), deep blue lasers (430 nm, 458 nm), which are necessary for optimal excitation of cyan fluorophores such as CFP, cerulean or turquoise, are much less common. Therefore, we list lasers suitable for ideal (one-photon) excitation in the figurative summaries. In addition to confocal microscopy, an increasing number of laboratories use two-photon microscopy, in particular, to study glial Ca^2+^ signaling *in vivo* (Srinivasan et al., 2015; Brawek et al., 2017; Stobart et al., 2018; Tran et al., 2018; Lines et al., 2020; Oe et al., 2020). Most of the GECIs are efficiently excited using a Ti:Sapphire laser in the range of 840 nm (e.g., TN-XXL) to 1,060 nm (e.g., jRGECO1a), although longer wavelengths up to 1,300 nm are preferred for 2-photon excitation of most “red” GECIs (Mohr et al., 2020).

### Glia-Specific Expression of GECIs

Before imaging Ca^2+^ one also has to have the GECI of choice expressed in astrocytes or glial cells of choice. Using viral vectors to express the GECI is highly effective. Recombinant AAV vectors are convenient and can be used at biosafety level 1 with the appropriate permissions. Many of the GECIs listed are available as plasmids with glial-specific promoters such as GFAP and gfaABC1D or GLAST that can be packaged into rAAVs with appropriate serotypes such as AAV8 or AAV9. Alternatively, breeding transgenic mice with GECIs encoded in their genome is a convenient and effective way to express GECIs in astrocytes for *in situ* and *in vivo* studies (Madisen et al., 2015; Sato et al., 2015; Bethge et al., 2017). Additional advantages of using transgenic mice are: (I) expression is non-invasive; (II) protein expression levels are usually more uniform; and (III) expression is usually lower than following rAAV transduction and hence the effect of additional Ca^2+^ buffering capacity is less severe. The disadvantage is the limited choice of GECI mouse lines. Members of the GCaMP6 family of GECIs, e.g., are commercially available as “floxed” mouse lines and can be cross-bred with mice that express Cre recombinase in astrocytes to achieve astrocyte-specific expression of GECIs (Madisen et al., 2015). Several astrocyte-specific Cre driver lines are available, which might differ in expression rate and specificity for certain brain regions. Cell-type specificity is achieved by using astrocyte-specific promoters to drive Cre expressions, such as Aldh1L1, GFAP, and GLAST (Yu et al., 2020). However, neuronal and oligodendroglial precursor cells may also express these astrocyte-typical proteins and hence Cre recombinase when using constitutively active promoters, resulting in GECI expression in cells derived from these precursors. This fate mapping effect can be circumvented by induction of GECI expression after neuronal and oligodendroglial maturation in inducible Cre driver mouse lines (tamoxifen or tetracycline/doxycycline). For some GECIs, transgenic mouse lines to allow for glia-specific Ca^2+^ imaging have been published, but are not commercially available (Yoshikawa et al., 2016; Egbert et al., 2019). According to our knowledge, we indicate whether transgenic mice, rAAVs, or plasmids ready-to-use for astrocytic expression are available and listed those in Supplementary Table 1.

### Combinability With Channelrhodopsins

Channelrhodopsins are light-activated ion channels used to control neuronal excitability (Nagel et al., 2003; Boyden et al., 2005). ChR2 is still the most commonly used optogenetic tool to excite neurons and hence is frequently employed to drive neuron-to-glia communication (Bernardinelli et al., 2011; Losi et al., 2017; Mariotti et al., 2018; Nikolic et al., 2018). As ChR2 is activated by blue light, it cannot be used together with GFP-based or CFP-based GECIs in widefield or confocal Ca^2+^ imaging experiments. However, they can be combined in two-photon Ca^2+^ imaging, since the spatially restricted stimulation of ChR2 is insufficient to significantly stimulate neurons (Losi et al., 2019). In widefield and confocal microscopy, violet-light-activated channels such as eTsChR (*Tetraselmis striata* channelrhodopsin) are better options (Farhi et al., 2019) or using ChR2 together with a red fluorescent GECI that is activated by green/yellow light. Some of these red fluorescent GECIs, however, use mApple as fluorophore, which photoswitches from a dim to a bright state upon illumination with blue light as used to stimulate ChR2, resulting in a Ca^2+^-independent fluorescence increase that interferes with the Ca^2+^ measurement (Akerboom et al., 2013; Dana et al., 2016).

## Recent Additions to The Ca^2+^ Indicator Portfolio

While we have limited the indicators presented in Figures 1, 2 to published calcium indicators tested in glial cells, there are some very interesting indicators published in the last few years that we think warrant mentioning. The blue to red X-CaMP series is interesting for multi-color imaging (Inoue et al., 2019). Still unpublished but already available on Addgene is the jGCaMP8 series from Janelia farms^1^). The K_d_ range from 46 nM (jGCaMP8s, “s” for sensitive) to 334 nM (jGCaMP8f, “f” for fast), and the slowest of these is as fast as GCaMP6f. None rival the apo brightness of jGCaMP7b, although resting brightness will also depend on resting Ca^2+^ concentration. Also of interest are jYCaMP1 and jYCaMP1s (K_d_ 79 and 70 nM) and XCaMP-Y, which are particularly suitable for 2-photon excitation using inexpensive pulsed lasers with a fixed wavelength around 1,030 nm (Inoue et al., 2019; Mohr et al., 2020). K-GECO is the first of a new series of bright red Ca^2+^ indicators designed to retain the excellent responsiveness of the R-GECO series and reduce photoswitching, which limits the usefulness of i.e., jRGECO1a (Shen et al., 2018). Ideal for confocal or widefield camera-based imaging, particularly in tissue, may be the near-infrared indicators NIR-GECO2 and NIR-GECO2G excited at 640 nm, although photobleaching remains problematic with long exposures (Qian et al., 2020). Very fast green and red indicators have also been developed but signals are smaller and to our knowledge, these have not been tested in astrocytes (Kerruth et al., 2019).

## Concluding Statement

There is not a single GECI that provides optimal characteristics for all applications. For quantification of changes in cytosolic Ca^2+^, FRET sensors are the method of choice, however, transgenic mouse models and ready-to-use viruses are not commercially available. Among the FRET sensors, Twitch-2B is a good choice and has been reported to work in non-excitable cells such as microglial cells (Brawek et al., 2017). Compared to FRET sensors, recent single-fluorophore sensors have a larger dynamic range and faster kinetics. Hence, they are more often used in glial cell research. Both transgenic mice and plasmids/viruses are available for the GCaMP6 family of GECIs, making them the first choice when a straightforward approach is pursued. The high dynamic range of GCaMP6s comes at the cost of very dim fluorescence at resting Ca^2+^ concentrations, hence GCaMP6f is favored as it allows visualization of astrocytes at rest. We have recently found that the single-wavelength sensor jGCaMP7b possesses an excellent combination of properties for use in astrocytes, including large dynamic range, fast kinetics, and high resting fluorescence and therefore included it in Figure 1. However, studies using jGCaMP7b in astrocytes have not been published yet and transgenic mice are not available to date.

## Author Contributions

All authors contributed to the research that is the base for this review as well as to the writing and editing of the manuscript. All authors contributed to the article and approved the submitted version.

## Conflict of Interest

The authors declare that the research was conducted in the absence of any commercial or financial relationships that could be construed as a potential conflict of interest.

## Footnotes

^1^https://www.janelia.org/jgcamp8-calcium-indicators
