# Supplementary material for: Using Genetically Encoded Calcium Indicators to Study Astrocyte Physiology: A Field Guide
Source: Front Cell Neurosci. 2021 Jun 11;15:690147. doi: 10.3389/fncel.2021.690147 (PMC8226001; doi:10.3389/fncel.2021.690147)
Supplement: Supplementary file 1 [file Table_1.pdf]

Supplementary Material

Supplementary table 1. List of available genetic tools for using GECIs in astrocytes

Mouse models

Ca^{2+} indicator	Allele	Source	Stock number
GCaMP6f (Cre-dependent)[1]	B6J.Cg-Gt(ROSA)26Sortm95.1(CAG-GCaMP6f)Hze/MwarJ	Jax	028865
GCaMP6s (Cre-dependent)[1]	B6J.Cg-Gt(ROSA)26Sortm96(CAG-GCaMP6s)Hze/MwarJ	Jax	028866
GCaMP6s (tetO-dependent)[2]	B6;DBA-Tg(tetO-GCaMP6s)2Niell/J	Jax	024742
GCaMP6f (Cre-dependent; targeted to the plasma membrane)[1]	C57BL/6N-Gt(ROSA)26Sortm1(CAG-GCaMP6f)Khakh/J	Jax	029626
G-CaMP7 (tetO-dependent)[2]	C57BL/6J-Tg(tetO-G-CaMP7,-DsRed2)572Bsi	Riken	RBRC06510
G-CaMP7 (Slc1a2-dependent)	C57BL/6-Tg(Slc1a2-G-CaMP7)Bsi	Riken	RBRC09650
R-CaMP1.07 (tetO-dependent)[2]	Igs7tm143.1(tetO-RCaMP1.07)Hze	Jax	030217
YC-nano50 (tetO-dependent)[2]	B6;129-Actb<tm2.1(tetO-YCnano50)Kftnk>	Riken	RBRC09550

Viruses (ready-to-use for astrocyte research)

Ca^{2+} indicator	Allele	Virus	Source	Virus number
GCaMP6f	pZac2.1 gfaABC1D-cyto-GCaMP6f	AAV5	Addgene	52925-AAV5
GCaMP6f (targeted to the plasma membrane)	pZac2.1 gfaABC1D-lck-GCaMP6f	AAV5	Addgene	52924-AAV5

Plasmids (ready-to-use for astrocyte research)

Ca^{2+} indicator	Allele	Source	Plasmid number
GCaMP6f	pZac2.1 gfaABC1D-cyto-GCaMP6f	Addgene	52925
GCaMP6f (targeted to the plasma membrane)	pZac2.1 gfaABC1D-lck-GCaMP6f	Addgene	52924
R-CaMP1.07	pAAV-hGfap-R-CaMP1.07-WPRE-SV40	Addgene	164140
jGCaMP7b	pAAV-GFAP-jGCaMP7b	Addgene	171118
jRCaMP1a	pAAV-gfaABC1D-NES-jRCaMP1a	Addgene	171120

[1]requires astrocyte-specific Cre driver mouse line; [2]requires astrocyte-specific tetO driver mouse line
